# (3-{[*N*-(5-Chloro-2-hy­droxy­phen­yl)oxamo­yl]amino}­prop­yl)dimethyl­aza­nium perchlorate

**DOI:** 10.1107/S1600536811050653

**Published:** 2011-12-03

**Authors:** Xi-Teng Yue, Xiao-Wen Li, Zhi-Yong Wu

**Affiliations:** a College of Feixian, Linyi University, 273400 Linyi, Shandong, People’s Republic of China; b Marine Drug and Food Institute, Ocean University of China, 266003 Qingdao, People’s Republic of China

## Abstract

In the title compound, C_13_H_19_ClN_3_O_3_^+^·ClO_4_^−^, the 3-(di­meth­yl­ammonio)­propyl group of the cation is disordered over two sets of sites with occupancies 0.772 (6) and 0.228 (6). The cations are joined by pairs of N—H⋯O hydrogen bonds into centrosymmetric dimers and these dimers are assembled into chains along the *a*-axis direction, also through N—H⋯O hydrogen bonds. The perchlorate anions are linked to the hy­droxy groups of the cations by O—H⋯O hydrogen bonds. The positively charged ammonium groups and the anions give rise to folded layers parallel to the *ab* plane.

## Related literature

For DNA binding of oxamide complexes, see: Li *et al.* (2010 ▶). For the synthesis, see: Tao *et al.* (2003 ▶).
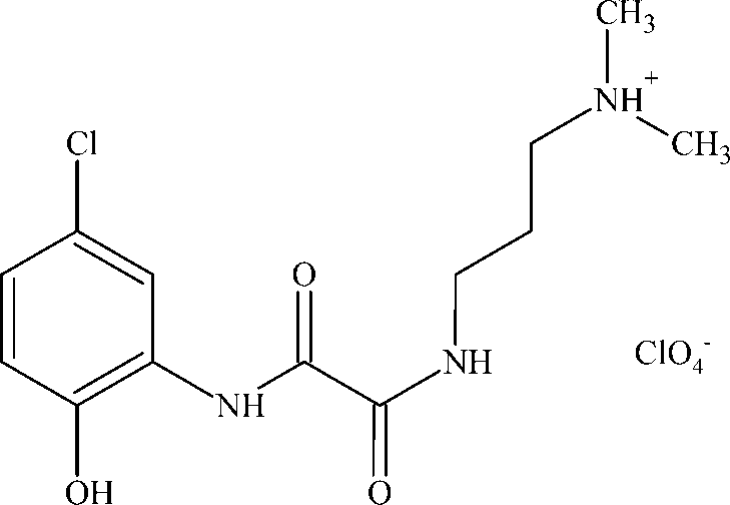


## Experimental

### 

#### Crystal data

C_13_H_19_ClN_3_O_3_^+^·ClO_4_^−^
*M* *_r_* = 400.21
Monoclinic, 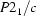

*a* = 6.7423 (5) Å
*b* = 12.8169 (10) Å
*c* = 21.6454 (17) Å
β = 98.275 (1)°
*V* = 1851.0 (2) Å^3^
*Z* = 4
Mo *K*α radiation
μ = 0.39 mm^−1^
*T* = 296 K
0.52 × 0.28 × 0.13 mm

#### Data collection

Bruker APEX area-detector diffractometer
Absorption correction: multi-scan (*SADABS*; Bruker, 2002 ▶) *T*_min_ = 0.823, *T*_max_ = 0.951
10727 measured reflections
4204 independent reflections
2526 reflections with *I* > 2σ(*I*)
*R*_int_ = 0.028

#### Refinement

*R*[*F* ^2^ > 2σ(*F* ^2^)] = 0.054
*wR*(*F* ^2^) = 0.179
*S* = 1.03
4204 reflections
297 parameters
16 restraints
H atoms treated by a mixture of independent and constrained refinement
Δρ_max_ = 0.56 e Å^−3^
Δρ_min_ = −0.26 e Å^−3^

### 

Data collection: *SMART* (Bruker, 2002 ▶); cell refinement: *SAINT* (Bruker, 2002 ▶); data reduction: *SAINT*; program(s) used to solve structure: *SHELXS97* (Sheldrick, 2008 ▶); program(s) used to refine structure: *SHELXL97* (Sheldrick, 2008 ▶); molecular graphics: *XP* in *SHELXTL* (Sheldrick, 2008 ▶); software used to prepare material for publication: *WinGX* (Farrugia, 1999 ▶).

## Supplementary Material

Crystal structure: contains datablock(s) I, global. DOI: 10.1107/S1600536811050653/yk2031sup1.cif

Structure factors: contains datablock(s) I. DOI: 10.1107/S1600536811050653/yk2031Isup2.hkl

Supplementary material file. DOI: 10.1107/S1600536811050653/yk2031Isup3.cml

Additional supplementary materials:  crystallographic information; 3D view; checkCIF report

## Figures and Tables

**Table 1:** Hydrogen-bond geometry (Å, °)

*D*—H⋯*A*	*D*—H	H⋯*A*	*D*⋯*A*	*D*—H⋯*A*
O1—H1⋯O5	0.89 (4)	1.80 (4)	2.693 (3)	174 (3)
N2—H2*A*⋯O2^i^	0.88 (3)	2.12 (3)	2.905 (3)	149 (2)
N3*A*—H3*A*⋯O3^ii^	0.91	2.04	2.814 (6)	142
N3*B*—H3*B*⋯O3^ii^	0.91	2.13	2.905 (18)	143

Symmetry codes: (i) 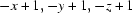; (ii) 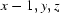.

**Fig. 1.**
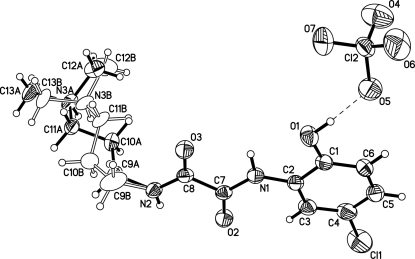
The structure of the title compound. The displacement ellipsoids are drawn at the 30% probability levels and H atoms are shown as small spheres of arbitrary radii. Two position of a disordered group are depicted in different styles. Dotted line indicate hydrogen bond.

**Fig. 2.**
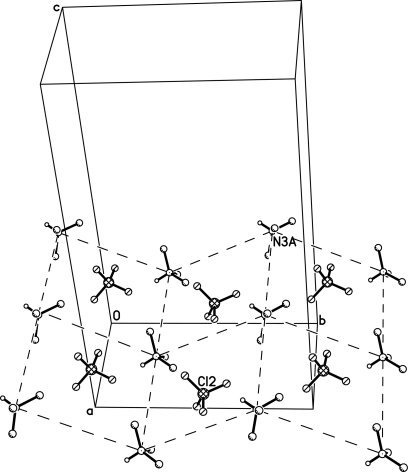
A folded layer structure parallel to a0b plane formed by the positively charged ammonium groups and the perchlorate ions with a quadrilateral pattern.

**Fig. 3.**
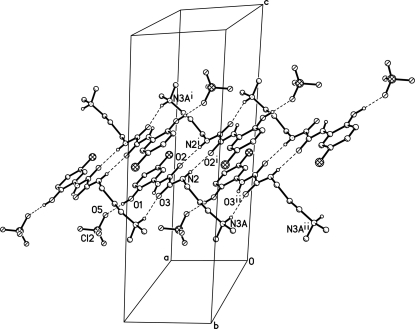
A view of a hydrogen bonding in the title compound. Symmetry codes: i = -x + 1, -y + 1, -z + 1; ii = x - 1, y, z.

## supplementary crystallographic information

### Comment

Aimed for the DNA-binding study of a series of asymmetric *N*,*N*-disubstituted oxamide complexes (Li *et al.*, 2010), we attempted to prepare a binuclear copper(II) complex with *N*-(5-chloro-2-hydroxyphenyl)-*N*'-(3-(dimethylammonio)propyl) oxamide (H_3_chdpoxd). Unexpectedly, the perchlorate salt of the ligand, (H_4_chdpoxd)ClO_4_, I, was obtained.

The title compound consists of a H_4_chdpoxd^+^ cations and a ClO4^-^ anions (Fig. 1). In the cation, the oxamide group adopts transoid conformation. The benzene ring is nearly parallel to the oxamide plane, with a dihedral angle of 5.39 (15)°. As for the alkyl substituent, the torsion angles of C8—N2—C9A—C10A and C8—N2—C9B—C10B are 86.1 (6)° and 117.6 (19)°, respectively.

In the crystal, the positively charged ammonium N atoms (N3A and N3B) together with the perchlorate anions form a folded layer structure with a quadrilateral pattern  (Fig. 2). Such a charge-balanced layer is paralled to *a0b* plane. Cations related by an inversion center are linked by the hydrogen bonds involving oxamide groups (N2—H2A···O2, Table 3) to form a dimer. These dimers form chains parallel to *a* direction through the hydrogen bonds involving the ammonium groups (Fig. 3). The perchlorate ions append to the chains through the hydrogen bonds with phenolic hydroxy groups.

### Experimental

The ligand, H_3_chdpoxd, was prepared according to the method proposed by Tao *et al.*, (2003). To a solution of H_3_chdpoxd (0.0299 g, 0.1 mmol) in methanol (10 ml) were added sequentially piperidine (0.2 mmol) and a solution of Cu(ClO_4_)_2_.6H_2_O (0.0742 g, 0.2 mmol) in methanol (10 ml). The mixture was intensively stirred until the solution became clear, and then 2,9-dimethyl-1,10-phenanthroline (dmphen, 0.0432 g, 0.4 mmol) in methanol (10 ml) was added. Stirring of the reaction mixture was continued at 333 K for 2 h. Although our original goal was to prepare a dinuclear copper(II) complex with chdpoxd^3-^ as a bridge ligand and 2,9-dimethyl-1,10-phenanthroline as a terminal ligand, the colourless crystals of the title compound, (H_4_chdpoxd)ClO_4_, unexpectedly precipitated on the seventh day, after the solution had been left to stand at room temperature.

Anal. Calcd for C_13_H_19_Cl_2_N_3_O_7_ (%): C, 39.01; H, 4.79; N, 10.50. Found: C, 38.49, H, 4.72, N, 11.06.

### Refinement

The 3-(dimethylamminio)propyl group of the cation is disordered over two sets of positions, suffixed with A and B. The occupancies were refined freely to 0.772 (6) and 0.228 (6), respectively. The bond lengths C9A—C10A, C10A—C11A and C10B—C11B were restrained to 1.54 Å with DFIX instructionto to avoid the unreasonable geometries. The H atoms on the phenolic hydroxyl and the oxamide group were found in a difference Fourier map and then refined freely except for the restrain on N1—H1A bond length of 0.86 Å. Other H atoms were placed in calculated positions, with C—H = 0.93 (aromatic), 0.97 (methylene) and 0.96 (methyl) and N—H = 0.91 Å, and refined using riding model, with *U*_iso_(H) = 1.2 *U*_eq_, or 1.5 *U*_eq_ for methyl groups.

### Crystal data

**Table:**

C_13_H_19_ClN_3_O_3_^+^·ClO_4_^−^	*F*(000) = 832
*M**_r_* = 400.21	*D*_x_ = 1.436 Mg m^−^^3^
Monoclinic, *P*2_1_/*c*	Mo *K*α radiation, λ = 0.71073 Å
Hall symbol: -P 2ybc	Cell parameters from 2302 reflections
*a* = 6.7423 (5) Å	θ = 2.5–23.6°
*b* = 12.8169 (10) Å	µ = 0.39 mm^−^^1^
*c* = 21.6454 (17) Å	*T* = 296 K
β = 98.275 (1)°	Block, colourless
*V* = 1851.0 (2) Å^3^	0.52 × 0.28 × 0.13 mm
*Z* = 4	

### Data collection

**Table:**

Bruker APEX area-detector diffractometer	4204 independent reflections
Radiation source: fine-focus sealed tube	2526 reflections with *I* > 2σ(*I*)
graphite	*R*_int_ = 0.028
φ and ω scans	θ_max_ = 27.6°, θ_min_ = 1.9°
Absorption correction: multi-scan (*SADABS*; Bruker, 2002)	*h* = −8→7
*T*_min_ = 0.823, *T*_max_ = 0.951	*k* = −16→16
10727 measured reflections	*l* = −28→27

### Refinement

**Table:**

Refinement on *F*^2^	Primary atom site location: structure-invariant direct methods
Least-squares matrix: full	Secondary atom site location: difference Fourier map
*R*[*F*^2^ > 2σ(*F*^2^)] = 0.054	Hydrogen site location: inferred from neighbouring sites
*wR*(*F*^2^) = 0.179	H atoms treated by a mixture of independent and constrained refinement
*S* = 1.03	*w* = 1/[σ^2^(*F*_o_^2^) + (0.0933*P*)^2^ + 0.3342*P*] where *P* = (*F*_o_^2^ + 2*F*_c_^2^)/3
4204 reflections	(Δ/σ)_max_ < 0.001
297 parameters	Δρ_max_ = 0.56 e Å^−^^3^
16 restraints	Δρ_min_ = −0.26 e Å^−^^3^

### Special details

**Table:**

Geometry. All s.u.'s (except the s.u. in the dihedral angle between two l.s. planes) are estimated using the full covariance matrix. The cell s.u.'s are taken into account individually in the estimation of s.u.'s in distances, angles and torsion angles; correlations between s.u.'s in cell parameters are only used when they are defined by crystal symmetry. An approximate (isotropic) treatment of cell s.u.'s is used for estimating s.u.'s involving l.s. planes.
Refinement. Refinement of *F*^2^ against ALL reflections. The weighted *R*-factor *wR* and goodness of fit *S* are based on *F*^2^, conventional *R*-factors *R* are based on *F*, with *F* set to zero for negative *F*^2^. The threshold expression of *F*^2^ > σ(*F*^2^) is used only for calculating *R*-factors(gt) *etc*. and is not relevant to the choice of reflections for refinement. *R*-factors based on *F*^2^ are statistically about twice as large as those based on *F*, and *R*- factors based on ALL data will be even larger.

### Fractional atomic coordinates and isotropic or equivalent isotropic displacement parameters (Å^2^)

**Table:**

	*x*	*y*	*z*	*U*_iso_*/*U*_eq_	Occ. (<1)
Cl1	1.0226 (3)	0.10172 (8)	0.45657 (6)	0.1407 (6)	
O1	1.2592 (3)	0.49690 (16)	0.35643 (11)	0.0730 (6)	
O2	0.7001 (3)	0.45689 (16)	0.47116 (10)	0.0706 (6)	
O3	0.8146 (3)	0.69101 (15)	0.40187 (9)	0.0674 (5)	
N1	0.9394 (3)	0.49367 (17)	0.41071 (10)	0.0555 (5)	
C1	1.2142 (4)	0.4024 (2)	0.37909 (12)	0.0548 (6)	
C2	1.0414 (4)	0.39935 (19)	0.40791 (11)	0.0521 (6)	
C3	0.9810 (5)	0.3065 (2)	0.43123 (13)	0.0663 (8)	
H3	0.8656	0.3031	0.4501	0.080*	
C4	1.0967 (6)	0.2179 (2)	0.42594 (15)	0.0814 (10)	
C5	1.2658 (6)	0.2206 (2)	0.39791 (14)	0.0791 (9)	
H5	1.3401	0.1603	0.3947	0.095*	
C6	1.3251 (5)	0.3137 (2)	0.37437 (13)	0.0665 (8)	
H6	1.4401	0.3164	0.3553	0.080*	
C7	0.7847 (3)	0.5170 (2)	0.43988 (11)	0.0488 (6)	
C8	0.7207 (4)	0.6308 (2)	0.43042 (11)	0.0499 (6)	
N2	0.5596 (3)	0.65522 (19)	0.45564 (10)	0.0549 (5)	
C9A	0.4556 (10)	0.7539 (4)	0.4449 (3)	0.0573 (15)	0.772 (6)
H9A	0.5504	0.8093	0.4403	0.069*	0.772 (6)
H9B	0.3859	0.7706	0.4798	0.069*	0.772 (6)
C10A	0.3053 (5)	0.7430 (3)	0.3848 (2)	0.0621 (11)	0.772 (6)
H10A	0.3783	0.7337	0.3498	0.075*	0.772 (6)
H10B	0.2245	0.6811	0.3879	0.075*	0.772 (6)
C11A	0.1694 (6)	0.8358 (3)	0.37263 (17)	0.0574 (11)	0.772 (6)
H11A	0.1131	0.8526	0.4102	0.069*	0.772 (6)
H11B	0.2467	0.8954	0.3622	0.069*	0.772 (6)
N3A	0.0037 (8)	0.8157 (4)	0.3207 (2)	0.0601 (14)	0.772 (6)
H3A	−0.0604	0.7570	0.3308	0.072*	0.772 (6)
C12A	0.0819 (11)	0.7936 (7)	0.2601 (3)	0.0734 (19)	0.772 (6)
H12A	0.1502	0.8541	0.2477	0.110*	0.772 (6)
H12B	−0.0281	0.7768	0.2284	0.110*	0.772 (6)
H12C	0.1733	0.7358	0.2658	0.110*	0.772 (6)
C13A	−0.1473 (9)	0.9010 (5)	0.3133 (3)	0.0913 (19)	0.772 (6)
H13A	−0.2004	0.9103	0.3517	0.137*	0.772 (6)
H13B	−0.2540	0.8832	0.2807	0.137*	0.772 (6)
H13C	−0.0850	0.9646	0.3026	0.137*	0.772 (6)
C9B	0.482 (3)	0.7601 (11)	0.4581 (12)	0.098 (11)	0.228 (6)
H9C	0.5740	0.8071	0.4416	0.118*	0.228 (6)
H9D	0.4825	0.7783	0.5016	0.118*	0.228 (6)
C10B	0.2703 (17)	0.7816 (12)	0.4234 (5)	0.071 (4)	0.228 (6)
H10C	0.1797	0.7278	0.4340	0.085*	0.228 (6)
H10D	0.2233	0.8481	0.4371	0.085*	0.228 (6)
C11B	0.2654 (18)	0.7841 (11)	0.3535 (5)	0.064 (4)	0.228 (6)
H11C	0.3377	0.8453	0.3427	0.077*	0.228 (6)
H11D	0.3351	0.7231	0.3411	0.077*	0.228 (6)
N3B	0.061 (2)	0.7862 (15)	0.3176 (6)	0.059 (5)	0.228 (6)
H3B	−0.0068	0.7323	0.3324	0.071*	0.228 (6)
C12B	0.067 (4)	0.763 (2)	0.2495 (8)	0.109 (13)	0.228 (6)
H12D	0.1776	0.7996	0.2359	0.164*	0.228 (6)
H12E	−0.0557	0.7849	0.2251	0.164*	0.228 (6)
H12F	0.0847	0.6892	0.2441	0.164*	0.228 (6)
C13B	−0.059 (3)	0.8810 (14)	0.3249 (9)	0.078 (6)	0.228 (6)
H13D	−0.0671	0.8921	0.3683	0.118*	0.228 (6)
H13E	−0.1916	0.8723	0.3024	0.118*	0.228 (6)
H13F	0.0036	0.9402	0.3085	0.118*	0.228 (6)
Cl2	1.62273 (11)	0.56840 (6)	0.24890 (4)	0.0702 (3)	
O4	1.5678 (6)	0.5285 (3)	0.18955 (14)	0.1317 (11)	
O5	1.5772 (4)	0.4944 (2)	0.29410 (13)	0.1029 (9)	
O6	1.8348 (4)	0.5793 (3)	0.25838 (18)	0.1327 (12)	
O7	1.5414 (6)	0.6642 (3)	0.25755 (16)	0.1554 (15)	
H1	1.365 (5)	0.491 (3)	0.3362 (15)	0.081 (10)*	
H1A	0.955 (5)	0.544 (2)	0.3857 (14)	0.107 (13)*	
H2A	0.501 (4)	0.601 (2)	0.4705 (13)	0.059 (8)*	

### Atomic displacement parameters (Å^2^)

**Table:**

	*U*^11^	*U*^22^	*U*^33^	*U*^12^	*U*^13^	*U*^23^
Cl1	0.2476 (16)	0.0511 (5)	0.1406 (10)	0.0012 (7)	0.0861 (10)	0.0201 (5)
O1	0.0726 (13)	0.0608 (12)	0.0946 (15)	−0.0003 (10)	0.0425 (12)	0.0074 (10)
O2	0.0703 (12)	0.0647 (12)	0.0842 (14)	0.0021 (9)	0.0365 (11)	0.0226 (10)
O3	0.0708 (12)	0.0570 (11)	0.0813 (13)	0.0003 (9)	0.0340 (10)	0.0185 (9)
N1	0.0588 (13)	0.0515 (13)	0.0598 (13)	−0.0011 (10)	0.0205 (11)	0.0115 (10)
C1	0.0622 (15)	0.0564 (15)	0.0465 (13)	−0.0016 (12)	0.0105 (12)	0.0009 (11)
C2	0.0611 (15)	0.0497 (14)	0.0460 (13)	0.0003 (11)	0.0094 (12)	0.0019 (11)
C3	0.089 (2)	0.0538 (16)	0.0597 (16)	−0.0027 (14)	0.0243 (15)	0.0055 (13)
C4	0.136 (3)	0.0487 (17)	0.0644 (18)	0.0009 (17)	0.030 (2)	0.0056 (13)
C5	0.120 (3)	0.0584 (18)	0.0604 (17)	0.0236 (18)	0.0167 (18)	−0.0043 (14)
C6	0.0768 (19)	0.0693 (19)	0.0543 (15)	0.0127 (15)	0.0123 (14)	−0.0092 (14)
C7	0.0443 (12)	0.0567 (15)	0.0457 (12)	−0.0036 (11)	0.0073 (10)	0.0086 (11)
C8	0.0474 (13)	0.0581 (14)	0.0444 (13)	−0.0010 (11)	0.0066 (11)	0.0090 (11)
N2	0.0482 (12)	0.0598 (14)	0.0583 (13)	0.0049 (10)	0.0139 (10)	0.0147 (11)
C9A	0.055 (4)	0.053 (3)	0.065 (3)	0.014 (2)	0.015 (2)	0.015 (2)
C10A	0.057 (2)	0.056 (2)	0.073 (3)	0.0112 (17)	0.009 (2)	0.001 (2)
C11A	0.060 (2)	0.050 (2)	0.064 (2)	0.0094 (17)	0.0180 (18)	0.0115 (17)
N3A	0.045 (3)	0.055 (3)	0.081 (3)	0.004 (2)	0.011 (2)	0.022 (2)
C12A	0.078 (4)	0.071 (4)	0.067 (3)	−0.015 (3)	−0.004 (3)	0.005 (3)
C13A	0.071 (4)	0.079 (4)	0.124 (5)	0.033 (3)	0.015 (3)	0.042 (3)
C9B	0.042 (11)	0.15 (2)	0.102 (19)	−0.002 (12)	0.004 (11)	0.062 (15)
C10B	0.067 (9)	0.081 (10)	0.070 (9)	0.012 (7)	0.030 (7)	0.024 (8)
C11B	0.071 (9)	0.049 (8)	0.073 (9)	0.011 (7)	0.010 (7)	0.034 (7)
N3B	0.048 (9)	0.061 (11)	0.067 (9)	0.005 (7)	0.007 (6)	0.037 (7)
C12B	0.15 (2)	0.10 (2)	0.076 (14)	−0.075 (18)	0.012 (13)	−0.003 (12)
C13B	0.070 (13)	0.057 (10)	0.115 (15)	0.032 (10)	0.034 (12)	0.037 (10)
Cl2	0.0760 (5)	0.0676 (5)	0.0745 (5)	0.0045 (3)	0.0358 (4)	0.0035 (3)
O4	0.178 (3)	0.127 (3)	0.0937 (19)	−0.032 (2)	0.032 (2)	−0.0124 (18)
O5	0.0856 (16)	0.113 (2)	0.119 (2)	0.0062 (14)	0.0467 (15)	0.0427 (16)
O6	0.0826 (17)	0.139 (3)	0.188 (3)	−0.0109 (17)	0.0595 (19)	0.024 (2)
O7	0.220 (4)	0.114 (3)	0.148 (3)	0.087 (3)	0.081 (3)	0.015 (2)

### Geometric parameters (Å, °)

**Table:**

Cl1—C4	1.733 (3)	N3A—C13A	1.487 (6)
O1—C1	1.357 (3)	N3A—C12A	1.507 (7)
O1—H1	0.89 (4)	N3A—H3A	0.9100
O2—C7	1.220 (3)	C12A—H12A	0.9600
O3—C8	1.221 (3)	C12A—H12B	0.9600
N1—C2	1.396 (3)	C12A—H12C	0.9600
N1—C7	1.329 (3)	C13A—H13A	0.9600
N2—C8	1.321 (3)	C13A—H13B	0.9600
C7—C8	1.527 (4)	C13A—H13C	0.9600
N1—H1A	0.857 (10)	C9B—C10B	1.537 (10)
C1—C6	1.372 (4)	C9B—H9C	0.9700
C1—C2	1.399 (4)	C9B—H9D	0.9700
C2—C3	1.377 (4)	C10B—C11B	1.508 (7)
C3—C4	1.392 (4)	C10B—H10C	0.9700
C3—H3	0.9300	C10B—H10D	0.9700
C4—C5	1.366 (5)	C11B—N3B	1.483 (10)
C5—C6	1.379 (4)	C11B—H11C	0.9700
C5—H5	0.9300	C11B—H11D	0.9700
C6—H6	0.9300	N3B—C13B	1.480 (10)
N2—C9B	1.447 (10)	N3B—C12B	1.513 (10)
N2—C9A	1.448 (4)	N3B—H3B	0.9100
N2—H2A	0.88 (3)	C12B—H12D	0.9600
C9A—C10A	1.535 (6)	C12B—H12E	0.9600
C9A—H9A	0.9700	C12B—H12F	0.9600
C9A—H9B	0.9700	C13B—H13D	0.9600
C10A—C11A	1.501 (4)	C13B—H13E	0.9600
C10A—H10A	0.9700	C13B—H13F	0.9600
C10A—H10B	0.9700	Cl2—O7	1.369 (3)
C11A—N3A	1.489 (6)	Cl2—O4	1.383 (3)
C11A—H11A	0.9700	Cl2—O6	1.422 (3)
C11A—H11B	0.9700	Cl2—O5	1.428 (2)
			
C1—O1—H1	110 (2)	H11A—C11A—H11B	108.0
C7—N1—C2	129.8 (2)	C13A—N3A—C11A	112.4 (4)
C7—N1—H1A	108 (3)	C13A—N3A—C12A	111.4 (5)
C2—N1—H1A	121 (3)	C11A—N3A—C12A	111.8 (5)
O1—C1—C6	124.0 (3)	C13A—N3A—H3A	107.0
O1—C1—C2	115.4 (2)	C11A—N3A—H3A	107.0
C6—C1—C2	120.5 (3)	C12A—N3A—H3A	107.0
C3—C2—N1	124.0 (2)	N2—C9B—C10B	117.7 (11)
C3—C2—C1	119.8 (2)	N2—C9B—H9C	107.9
N1—C2—C1	116.2 (2)	C10B—C9B—H9C	107.9
C2—C3—C4	118.4 (3)	N2—C9B—H9D	107.9
C2—C3—H3	120.8	C10B—C9B—H9D	107.9
C4—C3—H3	120.8	H9C—C9B—H9D	107.2
C5—C4—C3	121.9 (3)	C11B—C10B—C9B	112.3 (12)
C5—C4—Cl1	119.8 (3)	C11B—C10B—H10C	109.2
C3—C4—Cl1	118.3 (3)	C9B—C10B—H10C	109.2
C4—C5—C6	119.4 (3)	C11B—C10B—H10D	109.2
C4—C5—H5	120.3	C9B—C10B—H10D	109.2
C6—C5—H5	120.3	H10C—C10B—H10D	107.9
C1—C6—C5	119.9 (3)	N3B—C11B—C10B	114.2 (10)
C1—C6—H6	120.0	N3B—C11B—H11C	108.7
C5—C6—H6	120.0	C10B—C11B—H11C	108.7
O2—C7—N1	125.7 (2)	N3B—C11B—H11D	108.7
O2—C7—C8	122.1 (2)	C10B—C11B—H11D	108.7
N1—C7—C8	112.2 (2)	H11C—C11B—H11D	107.6
O3—C8—N2	125.2 (2)	C13B—N3B—C11B	116.1 (13)
O3—C8—C7	120.9 (2)	C13B—N3B—C12B	111.0 (12)
N2—C8—C7	113.8 (2)	C11B—N3B—C12B	110.6 (12)
C8—N2—C9B	124.0 (14)	C13B—N3B—H3B	106.1
C8—N2—C9A	123.2 (4)	C11B—N3B—H3B	106.1
C8—N2—H2A	113.7 (18)	C12B—N3B—H3B	106.1
C9B—N2—H2A	122 (2)	N3B—C12B—H12D	109.5
C9A—N2—H2A	120.9 (18)	N3B—C12B—H12E	109.5
N2—C9A—C10A	107.6 (3)	H12D—C12B—H12E	109.5
N2—C9A—H9A	110.2	N3B—C12B—H12F	109.5
C10A—C9A—H9A	110.2	H12D—C12B—H12F	109.5
N2—C9A—H9B	110.2	H12E—C12B—H12F	109.5
C10A—C9A—H9B	110.2	N3B—C13B—H13D	109.5
H9A—C9A—H9B	108.5	N3B—C13B—H13E	109.5
C11A—C10A—C9A	112.9 (3)	H13D—C13B—H13E	109.5
C11A—C10A—H10A	109.0	N3B—C13B—H13F	109.5
C9A—C10A—H10A	109.0	H13D—C13B—H13F	109.5
C11A—C10A—H10B	109.0	H13E—C13B—H13F	109.5
C9A—C10A—H10B	109.0	O7—Cl2—O4	113.6 (2)
H10A—C10A—H10B	107.8	O7—Cl2—O6	107.9 (3)
N3A—C11A—C10A	111.6 (3)	O4—Cl2—O6	107.4 (2)
N3A—C11A—H11A	109.3	O7—Cl2—O5	111.86 (19)
C10A—C11A—H11A	109.3	O4—Cl2—O5	109.62 (19)
N3A—C11A—H11B	109.3	O6—Cl2—O5	106.11 (18)
C10A—C11A—H11B	109.3		
			
C7—N1—C2—C1	−172.9 (3)	O2—C7—C8—N2	−4.1 (4)
C7—N1—C2—C3	7.4 (4)	N1—C7—C8—N2	176.1 (2)
O1—C1—C2—C3	178.7 (2)	O3—C8—N2—C9B	−6.1 (9)
C6—C1—C2—C3	−0.6 (4)	C7—C8—N2—C9B	173.8 (8)
O1—C1—C2—N1	−1.1 (3)	O3—C8—N2—C9A	9.3 (5)
C6—C1—C2—N1	179.7 (2)	C7—C8—N2—C9A	−170.8 (3)
N1—C2—C3—C4	−179.5 (3)	C8—N2—C9A—C10A	86.1 (6)
C1—C2—C3—C4	0.8 (4)	C9B—N2—C9A—C10A	−176 (8)
C2—C3—C4—C5	−0.7 (5)	N2—C9A—C10A—C11A	172.9 (4)
C2—C3—C4—Cl1	178.9 (2)	C9A—C10A—C11A—N3A	−170.2 (5)
C3—C4—C5—C6	0.4 (5)	C10A—C11A—N3A—C13A	172.5 (5)
Cl1—C4—C5—C6	−179.2 (2)	C10A—C11A—N3A—C12A	−61.4 (6)
O1—C1—C6—C5	−178.9 (3)	C8—N2—C9B—C10B	117.6 (19)
C2—C1—C6—C5	0.3 (4)	C9A—N2—C9B—C10B	27 (5)
C4—C5—C6—C1	−0.2 (5)	N2—C9B—C10B—C11B	−73 (3)
C2—N1—C7—O2	0.1 (4)	C9B—C10B—C11B—N3B	169.9 (13)
C2—N1—C7—C8	179.9 (2)	C10B—C11B—N3B—C13B	65.8 (18)
O2—C7—C8—O3	175.8 (2)	C10B—C11B—N3B—C12B	−166.5 (16)
N1—C7—C8—O3	−4.0 (3)		

### Hydrogen-bond geometry (Å, °)

**Table:**

*D*—H···*A*	*D*—H	H···*A*	*D*···*A*	*D*—H···*A*
O1—H1···O5	0.89 (4)	1.80 (4)	2.693 (3)	174 (3)
N2—H2A···O2^i^	0.88 (3)	2.12 (3)	2.905 (3)	149 (2)
N3A—H3A···O3^ii^	0.91	2.04	2.814 (6)	142.
N3B—H3B···O3^ii^	0.91	2.13	2.905 (18)	143.

Symmetry codes: (i) −*x*+1, −*y*+1, −*z*+1; (ii) *x*−1, *y*, *z*.
